# Cell cycle, energy metabolism and DNA repair pathways in cancer cells are suppressed by Compound Kushen Injection

**DOI:** 10.1186/s12885-018-5230-8

**Published:** 2019-01-24

**Authors:** Jian Cui, Zhipeng Qu, Yuka Harata-Lee, Thazin Nwe Aung, Hanyuan Shen, Wei Wang, David L. Adelson

**Affiliations:** 1 Department of Molecular and Biomedical Science, The University of Adelaide, North Terrace, Adelaide, 5005 South Australia Australia; 2 Zhendong Australia - China Centre for Molecular Chinese Medicine, The University of Adelaide, North Terrace, Adelaide, 5005 South Australia Australia; 3 Zhendong Research Institute, Shanxi-Zhendong Pharmaceutical Co Ltd, Beijing, China

**Keywords:** Alkaloid, Matrine, Cyclin, Ku70, Ku80, Cell-cycle

## Abstract

**Background:**

In this report we examine candidate pathways perturbed by Compound Kushen Injection (CKI), a Traditional Chinese Medicine (TCM) that we have previously shown to alter the gene expression patterns of multiple pathways and induce apoptosis in cancer cells.

**Methods:**

We have measured protein levels in Hep G2 and MDA-MB-231 cells for genes in the cell cycle pathway, DNA repair pathway and DNA double strand breaks (DSBs) previously shown to have altered expression by CKI. We have also examined energy metabolism by measuring [ADP]/[ATP] ratio (cell energy charge), lactate production and glucose consumption. Our results demonstrate that CKI can suppress protein levels for cell cycle regulatory proteins and DNA repair while increasing the level of DSBs. We also show that energy metabolism is reduced based on reduced glucose consumption and reduced cellular energy charge.

**Results:**

Our results validate these pathways as important targets for CKI. We also examined the effect of the major alkaloid component of CKI, oxymatrine and determined that it had no effect on DSBs, a small effect on the cell cycle and increased the cell energy charge.

**Conclusions:**

Our results indicate that CKI likely acts through the effect of multiple compounds on multiple targets where the observed phenotype is the integration of these effects and synergistic interactions.

**Electronic supplementary material:**

The online version of this article (10.1186/s12885-018-5230-8) contains supplementary material, which is available to authorized users.

## Background

Compound Kushen Injection (CKI) is a complex mixture of plant bioactives extracted from Kushen (*Sophora flavescens*) and Baituling (*Smilax Glabra*) that has been approved for use in China since 1995 by the State Food and Drug Administration of China (State medical license no. Z14021231).

Kushen has a long history in Chinese Medicine and is a very commonly used plant in the Chinese Materia Medica (CMM). This leguminous plant is widely distributed in Russia, Japan, India, North Korea, and some provinces and regions in mainland China [1]. The medicinal part of Kushen is its dry root that is used mainly to treat inflammation, eczema, parasites and similar afflictions [2, 3]. Modern pharmacological research suggests that the various alkaloids and flavonoids contained in Kushen have anticancer activity, especially with respect to arresting tumor growth and relieving cancer pain [4]. Compared to Kushen, Baituling is distributed in some regions of southern China, and was only used clinically in limited applications [5]. Because of this limited clinical usage, there are only a small number of research reports focused on Baituling.

The combination of above two herbs’ extracts, CKI is widely used in China as an adjunct for both radiotherapy and chemotherapy in cancer. While most of the data supporting its use have been anecdotal and there is little clinical trial data demonstrating its efficacy, it has been shown to be effective at reducing sarcoma growth and cancer pain in an animal model [6] and cancer pain in patients [4].

CKI contains over 200 chemical compounds including alkaloids and flavonoids such as matrine, oxymatrine and kurarinol, and has previously been shown to affect the cell cycle and induce apoptosis in cancer cells [2, 4, 6–10]. Furthermore, functional genomic characterisation of the effect of CKI on cancer cells using transcriptome data indicated that multiple pathways were most likely affected by CKI [8]. These observations support a model wherein many/all of the individual compounds present in CKI can act on many single targets or on multiple targets to induce apoptosis.

Based on previously reported work [8] and our currently unpublished work (Cui et al) [11], specific pathways were selected for follow up experiments to validate their response to CKI in order to formulate more specific hypotheses regarding the mechanism of action of CKI on cancer cells. We had previously shown that CKI altered the cell cycle and induced apoptosis while altering the expression of many cell cycle genes in three cancer cell lines [8, 11]. We had also shown that DNA repair pathway genes were significantly down-regulated by CKI and that energy production related to NAD(P)H synthesis from glycolysis and oxidative phosphorylation was reduced by CKI. As a result we focused on the following candidate pathways: cell cycle, DNA repair and glucose metabolism to validate their alteration by CKI. We used two cell lines for these validation experiments, one relatively insensitive to CKI (MDA-MB-231) and one sensitive to CKI (Hep G2). Furthermore, while the literature shows varying effects for major compounds present in CKI on cancer cells [12, 13], we also tested oxymatrine, the major alkaloid found in CKI and widely believed to be very important for the effects of CKI, on our selected pathways.

## Methods

### Cell culture and chemicals

CKI with a total alkaloid concentration of 26.5 mg/ml in 5 ml ampoules was provided by Zhendong Pharmaceutical Co. Ltd. (Beijing, China). Cell culture methods have been previously described [8]. The concentration of total alkaloids in CKI was determined using HPLC.

A human breast adenocarcinoma cell line, MDA-MB-231 and a hepatocellular carcinoma cell line Hep G2 were purchased from American Type Culture Collection (VA, USA). The cells were cultured in Dulbecco’s Modified Eagle Medium (Thermo Fisher Scientific, MA, USA) supplemented with 10% fetal bovine serum (Thermo Fisher Scientific). Both cell lines were cultured at 37 °C with 5% CO_2_. Cells were split twice per week with trypsinization, defined as two passages. Both cell lines were discarded when passage number was more than 15.

For all in vitro assays, cells were cultured overnight before being treated with CKI (either at 1 mg/ml and 2 mg/ml of total alkaloids). As a negative control, cells were treated with medium only and labelled as “untreated”. After 24 and 48 h of treatment, cells were harvested and subjected to the downstream experiments.

All the in vitro assays employed either 6-well plates or 96-well plates. The seeding density for 6-well plates for both cell lines was 4 ×10^5^ cells and treatment methods were as previously described [8]. The seeding density of Hep G2 cells for 96 well plates was 4 ×10^3^ cells per well and for MDA-MB-231 cells was 8 ×10^4^ cells per well, and used the same treatment method as above: after seeding and culturing overnight, cells were treated with 2 mg/ml CKI diluted with complete medium for the specified time.

### Glucose consumption assay

Glucose consumption was assessed in both cell lines in 6-well plates. Glucose consumption was determined by using a glucose oxidase test kit (GAGO-20, Sigma Aldrich, MO, USA). After culturing for different durations (3, 6, 12, 24 and 48 h), 50 *μ*l of culture medium was collected from untreated groups and treated groups. The cells were trypsinized for cell number determination using trypan blue exclusion assay and the number of bright, viable cells were counted using a hemocytometer. Collected suspension, blank medium and 2 mg/ml CKI, were all filtered and diluted 100 fold with MilliQ water. The absorbance at 560 nm was converted to glucose concentration using a 5 *μ*g/ml glucose standard from the kit as a single standard. Glucose consumption was calculated by subtracting the blank medium value from treated/collected medium values. Glucose consumption per cell was calculated from the number of cells determined above.

### Measurement of [ADP]/[ATP] ratio

Cells were cultured in white 96-well plates with clear bottoms. The [ADP]/[ATP] ratio of both cell lines was determined immediately after the incubation period (24 and 48 h) using an assay kit (MAK135; Sigma Aldrich) according to the manufacturer’s instructions. Levels of luminescence from the luciferase-mediated reaction was measured using a plate luminometer (PerkinElmer 2030 multilabel reader, MA, USA for CKI experiments or Promega, WI, USA for oxymatrine experiments). The [ADP]/[ATP] ratio was calculated from the luminescence values using a formula provided by the kit manufacturer.

### Lactate content assay

The concentration of lactate, the end product of glycolysis, was determined using a lactate colorimetric assay kit (Abcam, MA, USA). Cells were cultured in 6-well plates, and then harvested and deproteinized according to the manufacturer’s protocol. The optical density was measured at 450 nm and a standard curve plot (nmol/well vs. OD 450 nm) was generated using serial dilutions of lactate. Lactate concentrations were calculated with formula provided by the kit manufacturer.

### Cell cycle assay

Cells were cultured in 6-well plates and treated with 2 mg/ml CKI or 0.5 mg/ml oxymatrine. After culturing for 3, 6, 12, 24 and 48 h, cells were harvested and subjected to cell cycle analysis by propidium iodide staining as previously reported [8]. Data were obtained by flow cytometry using Accrui (BD Biosciences, NJ, USA) and analysed using FlowJo software (Tree Star Inc, OR, USA).

### Microscopy

After treating for 48 h on 22 ×22 Deckglaser cover glasses placed in 6-well plates, control and treated cells were fixed in 1% paraformaldehyde for 10 min at room temperature, and permeabilized with 0.5% Triton X-100 for 10 min. After fixation and permeabilization, cells were blocked with 5% fetal bovine serum for 30 min. Permeabilized cells were stained with 10 *μ*g/ml of Alexa Fluor$\circledR $594 conjugated anti- *γ*-H2AX Phospho (red) (BioLegend, CA, USA) in 5% fetal bovine serum overnight at 4 °C and mounted with 4’,6-diamidino-2-phenylindole (DAPI).

Stained cells were visualized with an Olympus FV3000 (Olympus Corporation, Tokyo, Japan) confocal microscope using a 20 × objective. Fluorescence intensity was automated pictured collection with ArrayScan XTI Live High Content Platform (Thermo Fisher Scientific) and software based nuclear analysis (HCS studio 3.0 Cell Analysis Software; Thermo Fisher Scientific) was implemented as for data acquirement of CircSpots under 20 × focusing. The acquired number of each cell line in one replicate was, approximately 5000 cells for Hep G2 and approximately 2000 cells for MDA-MB-231.

### Detection of intranuclear/intracellular proteins by flow cytometry

Cells were cultured in 6-well plates, treated with or without CKI, and harvested on different time points to detect intranuclear/intracellular levels of proteins involved in cell cycle, DNA repair and DNA DSBs pathways using the following antibodies. To measure levels of proteins involved in cell cycle, rabbit anti-CDK1 (Abcam), rabbit anti-p53, rabbit anti-cyclin D1, rabbit anti-CDK2 along with rabbit IgG isotype control (Cell Signalling Technologies, MA, USA) were used and these were detected with anti-rabbit IgG-PE (Cell Signalling Technologies). In addition, *β*-catenin levels were detected with rabbit anti- *β*-catenin-Alexa Fluor 647 with rabbit IgG-Alexa Fluor 647 isotype control (Abcam). The levels of proteins involved in DNA repair pathway were measured with rabbit anti-Ku70-Alexa Fluor 647 or with rabbit anti-Ku80-Alexa Fluor 647 (Abcam) along with rabbit IgG-Alexa Fluor 647 isotype control. For the detection of *γ*-H2AX involved in DSBs pathway, mouse anti- *γ*-H2AX-PE and mouse IgG1-PE isotype control (BioLegend) were used.

The cells prepared for the detection of cell cycle and DNA repair pathways were fixed and permeabilised using Nuclear Factor Fixation and Permeabilization Buffer Set (BioLegend) according to the manufacturer’s instructions. The cells prepared for the detection of DNA DSBs were fixed and permeabilised using chilled 70% ethanol. For samples under single time point and treatment, 2 ×10^5^ cells were labelled either with target antibody or corresponding isotype. The data was acquired on a FACS Canto (BD Biosciences, NJ, USA) or Accrui, and analysed using FlowJo software.

### Cell cycle functional enrichment re-analysis

In order to identify the phases of the cell cycle affected by CKI, differentially expressed gene data from Qu et al. and Cui et al. [8, 11] was submitted to the Reactome database [14], and used to identify functionally enriched genes.

### Statistical analysis

All assays above were performed in triplicate and repeated at least three times. Statistical significance was defined as *p*-value less than 0.05, and determined by *t*-test for microscopy and two-way ANOVA test for rest of the assays with GraphPad Prism (v7.03, Graphpad Software Inc., CA, USA); error bars represent standard deviation.

## Results

### Pathway validation

**Fig. 1:**
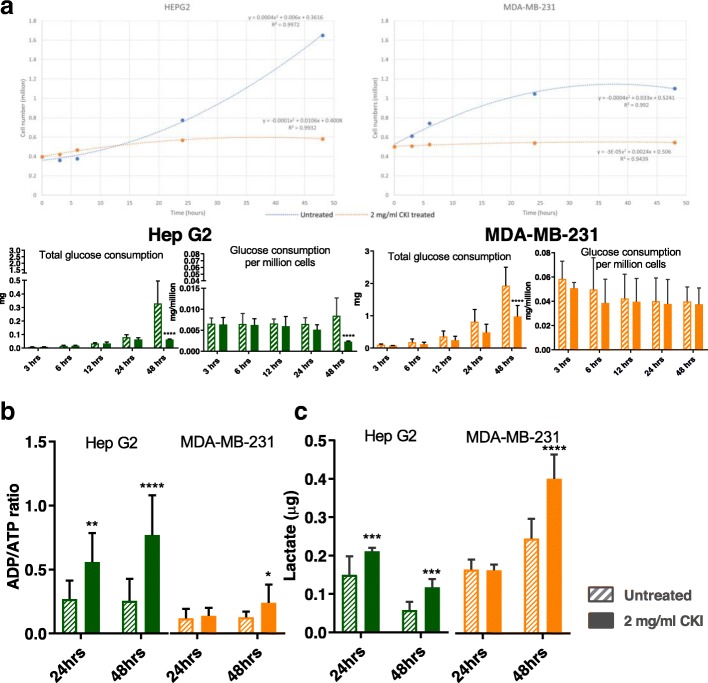
The energy metabolism determination assays in the two cell lines. **a** Growth curve (top panels) and comparison of glucose consumption analysis (lower panels) between the two cell lines at 3, 6, 12, 24 and 48 h. Overall glucose consumption is divided by cell number to calculate the consumption of glucose per million cells. **b** [ADP]/[ATP] ratio assay result for the two cell lines at 24 and 48 h. **c** Lactate content detection for the two cell lines at 24 and 48 h. Statistical analyses were performed using two-way ANOVA comparing treated with untreated (*p <0.05, **p <0.01, ***p <0.001, ****p <0.0001); bars show one standard deviation from the mean

Based on our previous results indicating that CKI could suppress NAD(P)H synthesis [8] and (Additional file 1: Figure S1), we examined the effect of CKI on energy metabolism by measuring glucose uptake, [ADP]/[ATP] ratio and lactate production. We measured glucose uptake in both CKI treated and untreated cells from 0 to 48 h after treatment and observed a reduction in glucose uptake (Fig. 1a). The growth curves for both cell lines were relatively flat after CKI treatment, in contrast to untreated cells. MDA-MB-231 cells, which are less sensitive to CKI in terms of apoptosis, had a higher level of glucose uptake than Hep G2 cells, which are more sensitive to CKI. Because the overall glucose uptake was consistent with the cell growth curves, the glucose consumption per million cells for each cell line under treatment was different. Untreated Hep G2 cells maintained a relatively flat rate of glucose consumption per million cells, while for CKI treated Hep G2 cells the rate of glucose consumption per million cells decreased with time, becoming significantly less towards 48 h. The glucose consumption variance for both untreated and treated MDA-MB-231 cells was high, but both overall glucose consumption and glucose consumption per million cells appeared to decrease over time.

Because changes in glucose consumption are mirrored by other aspects of energy metabolism, we assessed the energy charge of both CKI treated and untreated cells by measuring the [ADP]/[ATP] ratio at 24 and 48 h after treatment (Fig. 1b). Hep G2 cells had a lower energy charge (higher [ADP]/[ATP] ratio) compared to MDA-MB-231 cells and after CKI treatment both cell lines showed a decrease in energy charge, consistent with our previous measurements using a 2,3-Bis(2-methoxy-4-nitro-5-sulfonyl)-2H-tetrazolium-5-carboxanilideinner salt (XTT) assay (Additional file 1: Figure S1). However the decrease in energy charge was earlier and much more pronounced for Hep G2 cells compared to MDA-MB-231 cells.

The flip side of glucose consumption is the production of lactate via glycolysis, which is the initial pathway for glucose metabolism. We therefore measured lactate production in order to determine if the observed decreases in energy charge and glucose consumption were directly attributable to reduced glycolytic activity. We measured intracellular lactate concentration in both CKI treated and untreated cells at 24 and 48 h after treatment (Fig. 1c) and found that lactate concentrations increased as a function of CKI treatment in both cell lines. This result is consistent with a build up of lactate due to an inhibition of the Tricarboxylic Acid (TCA) cycle leading to decreased oxidative phosphorylation and lower cellular energy charge. CKI must therefore inhibit cellular energy metabolism downstream of glycolysis, most likely at the level of the TCA cycle. Decreased energy charge can have widespread effects on a number of energy hungry cellular processes involved in the cell cycle, such as DNA replication.

**Fig. 2:**
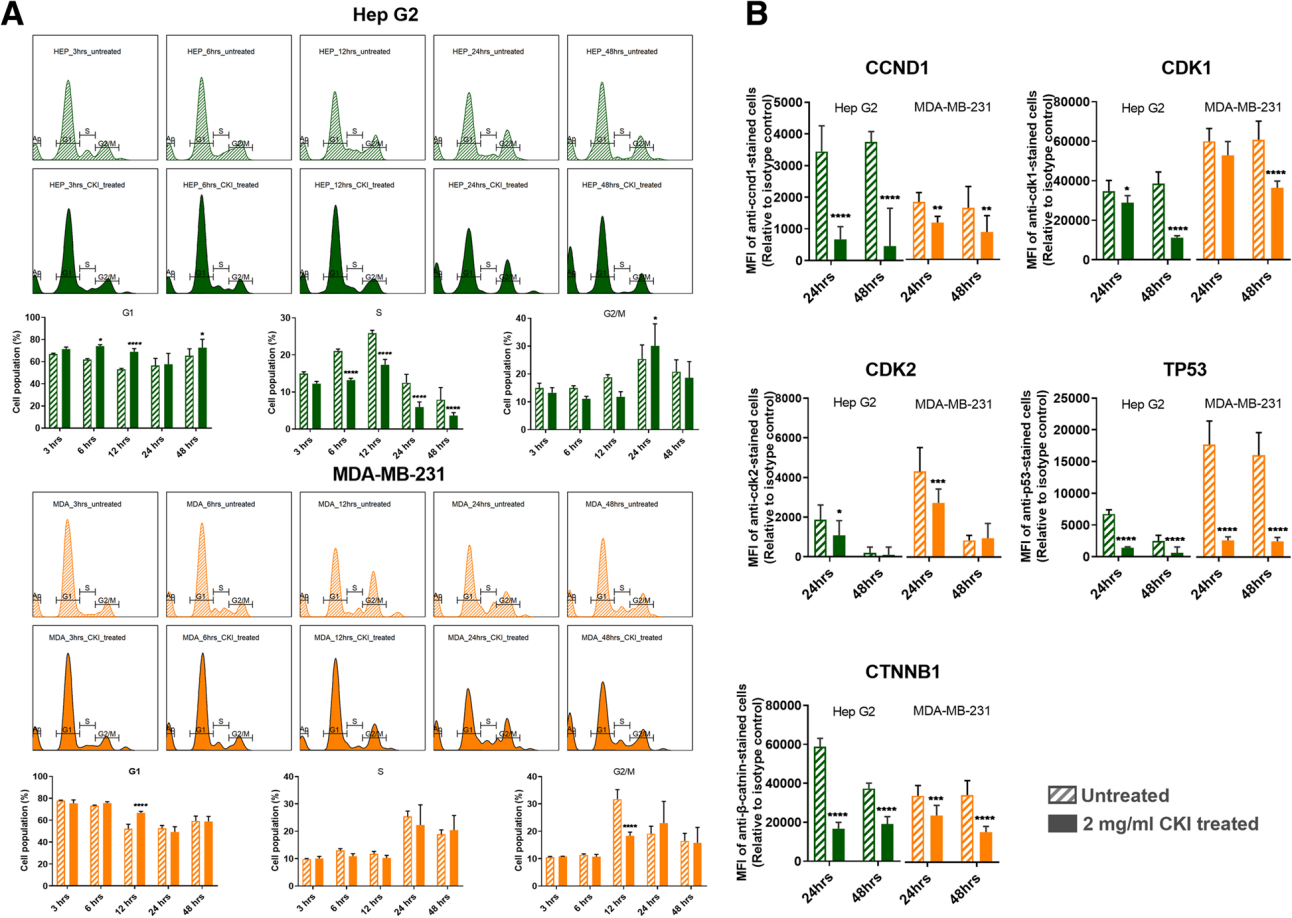
Cell cycle shift by CKI and changing expression of key proteins. **a** Histogram and statistical results of cell cycle shift regulated by CKI over 48 h. In both cell lines, the earliest shifted cell cycle phase was S phase 6 h after treatment. Compared to Hep G2, MDA-MB-231 showed delayed responses. **b** Expression levels for five proteins as a result of CKI treatment at both 24 and 48 h. Statistical analyses were performed using two-way ANOVA comparing treated with untreated (*p <0.05, **p <0.01, ***p <0.001, ****p <0.0001); bars show one standard deviation from the mean

Having validated the effect of CKI on cellular energy metabolism, we proceeded to examine the perturbation of cell cycle and expression of cell cycle proteins, as these are energy intensive processes. We had previously identified the cell cycle as a target for CKI based on transcriptome data from CKI treated cells [8, 11]. We carried out cell cycle profiling on CKI treated and untreated cells using propidium iodide staining and flow cytometry (Fig. 2a) as described in Materials and Methods. The two cell lines displayed slightly different profiles to each other, but their response to CKI was similar in terms of an increase in the proportion of cells in G1-phase. For Hep G2 cells, CKI caused consistent reductions in the proportion of cells in S-phase accompanied by corresponding increases in the proportion of cells in G1-phase. This is indicative of a block in S-phase leading to accumulation of cells in G1-phase. For MDA-MB-231 cells, CKI did not promote a significant decrease in the proportion of cells in S-phase, but did cause an increase in the percentage of cells in G1 phase at 24 h and a pronounced decrease in cells in G2/M phase at 12 h.

We also examined the levels of key proteins involved in the cell cycle pathway (Cyclin D1:CCND1, Cyclin Dependent Kinase 1:CDK1, Cyclin Dependent Kinase 2:CDK2, Tumor Protein p53:TP53 and Catenin Beta 1:CTNNB1) at 24 and 48 h after CKI treatment previously shown to have altered transcript expression by CKI (Fig. 2b). Both cell lines showed similar results for all five proteins, with decreased levels caused by CKI, and validated previous RNAseq data [8, 11]. CCND1 regulates the cell-cycle during G1/S transition. CDK-1 promotes G2-M transition, and regulates G1 progress and G1-S transition. CDK-2 acts at the G1-S transition to promote the E2F transcriptional program and the initiation of DNA synthesis, and modulates G2 progression. TP53acts to negatively regulate cell division. CTNNB1 acts as a negative regulator of centrosome cohesion. Down-regulation of these proteins is therefore consistent with cell cycle arrest/dysregulation and the cell cycle result in Fig. 2a. These results indicate that CKI alters cell cycle regulation consistent with cell cycle arrest. Cell cycle arrest is also an outcome that can result from DNA damage such as DSBs [15].

**Fig. 3:**
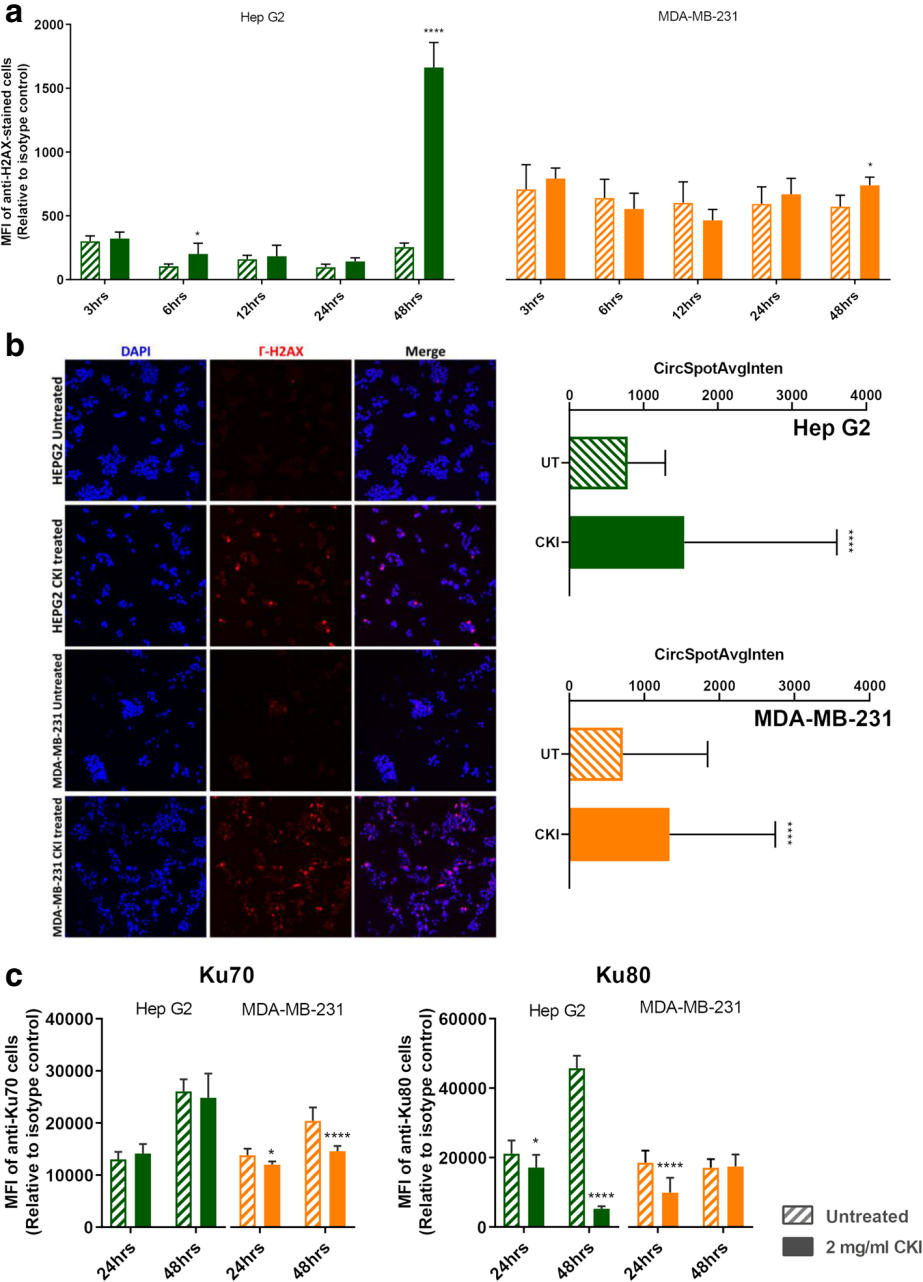
DSBs were increased by CKI treatment. **a** *γ*-H2AX expression from 3 to 48 h after treatment with 2 mg/ml CKI in two cell lines. **b** Localization of *γ*-H2AX in two cell lines after CKI treatment for 48 h. Blue is DAPI staining of nuclei, pink/red is staining of DSBs with antibody to *γ*-H2AX. The bar graph shows a quantification of the average number of *γ*-H2AX foci per cell detected in immunofluorescence images of 2 mg/ml CKI treated and untreated groups of 3 independent replicate experiments. **c** Expression of DSBs repair proteins, Ku70 and Ku80, as a result of treatment with 2 mg/ml CKI in two cell lines. Statistical analyses were performed using two-way ANOVA or *t*-test (for microscopy) comparing treated with untreated (*p <0.05, **p <0.01, ***p <0.001, ****p <0.0001); bars show one standard deviation from the mean

We had previously observed that DNA repair genes had lower transcript levels in CKI treated cells [8, 11], so hypothesised that this might result in increased numbers of DSBs. We measured the expression of *γ*-H2AX in both cell lines (Fig. 3a) and found that it was only over-expressed at 48 h in CKI treated Hep G2 cells. We also carried out localization of *γ*-H2AX using quantitative immunofluorescence microscopy and determined that the level of *γ*-H2AX increased in nuclei of CKI treated cells in both cell lines (Fig. 3b). These results indicated an increase in DSBs as a result of CKI treatment. In order to confirm if reduced expression of DNA repair proteins was correlated with the increase in DSBs we measured levels of Ku70/Ku80 proteins in CKI treated cells (Fig. 3c). In Hep G2 cells, Ku80, a critical component of the Non-Homologous End Joining (NHEJ) DNA repair pathway was significantly down-regulated at both 24 and 48 h after CKI treatment. In MDA-MB-231 cells, Ku70 expression was down-regulated at both 24 and 48 h after CKI treatment, and Ku80 was down-regulated at 24 h. Because Ku70/Ku80 are subunits of a required DNA repair complex, reduced expression of either subunit will result in decreased DNA repair. Our results therefore support a suppressive effect of CKI on DNA repair, likely resulting in an increased level of DSBs.

### Effect of oxymatrine, the principal alkaloid in CKI

Because CKI is a complex mixture of many plant secondary metabolites that may have many targets and there is little known about its molecular mode of action, we examined the effects of the most abundant single compound found in CKI, oxymatrine, on the most sensitive cell line, Hep G2. Oxymatrine is an alkaloid that has previously been reported to have effects similar to CKI, so we expected it might have an effect on one or more of our three validated pathways.

**Fig. 4:**
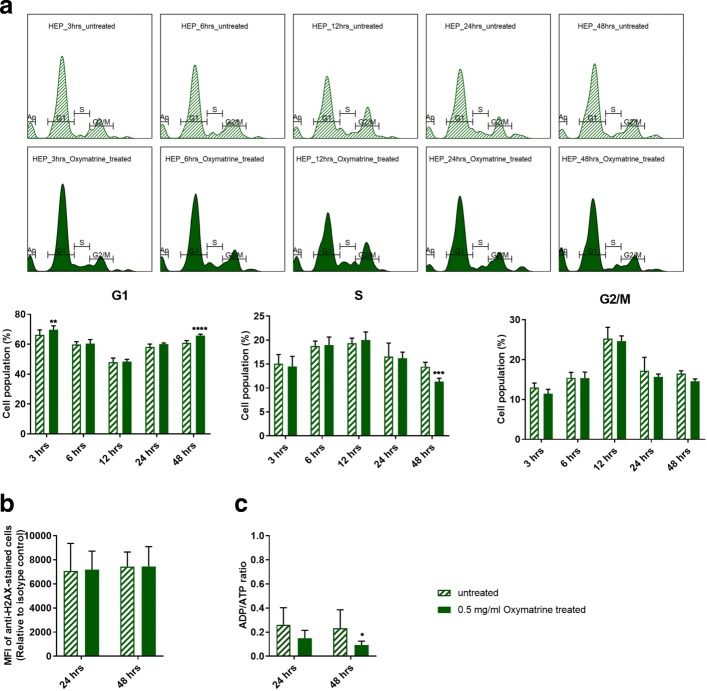
Effect of oxymatrine alone on validated pathways. Oxymatrine was tested at 0.5 mg/mL which is equivalent to its concentration in CKI. **a** Histogram and statistical results of cell cycle affected by oxymatrine over 48 h. **b** Effect of oxymatrine on *γ*-H2AX levels after 24 and 48 h. **c** Effect of oxymatrine on [ADP]/[ATP] ratio after 24 and 48 h. Statistical analyses were performed using two-way ANOVA comparing treated with untreated (*p <0.05, **p <0.01, ***p <0.001, ****p <0.0001); bars show one standard deviation from the mean

Oxymatrine, at 0.5 mg/ml which is equivalent to the concentration of oxymatrine in 2 mg/ml CKI, did not have an equivalent effect on the cell cycle compared to CKI (Fig. 4a vs Fig. 2a). Oxymatrine caused only minor changes to the cell cycle with small but significant increases in the proportion of cells in G1-phase at 3 and 48 h and a small but significant decrease in the proportion of cells in S1-phase at 48 h. Oxymatrine also caused a significant increase in the proportion of cells undergoing apoptosis in Hep G2 cells, albeit at a lower level than CKI (Additional file 1: Figure S2).

Oxymatrine had no effect on *γ*-H2AX levels in Hep G2 cells (Fig. 4b). This was in stark contrast to the effect of CKI (Fig. 3a) at 48 h and indicated that oxymatrine alone had no effect on the level of DSBs.

Surprisingly, oxymatrine had the opposite effect on energy metabolism compared to CKI, causing a decrease in [ADP]/[ATP] ratio indicating a large increase in the energy charge of the cells (Fig. 4c).

### Integration of results

The effect of CKI on cancer cells was validated in all three of our candidate pathways: cell cycle, energy metabolism and DNA repair. Because these pathways are not isolated, but instead are integrated aspects of cell physiology CKI may act through targets in some or all of these three pathways or may act through other targets that either directly or indirectly suppress these pathways. CKI may also act through the synergistic effects of multiple compounds on multiple targets in our candidate pathways. This possibility is consistent with the partial and minor effects of oxymatrine on our candidate pathways.

## Discussion

We have validated three pathways (cell cycle, energy metabolism and DNA repair) that are perturbed by CKI and that can be used as the focus for further investigations to identify specific molecular targets that mediate the perturbations.

### Cell cycle perturbation by CKI

Our results show that CKI can perturb the cell cycle by altering the proportions of cells in G1-phase, S-phase and G2/M-phase. This result is similar to what we have observed before [8, 11], but has not been widely reported in the literature. The alkaloid oxymatrine, the most abundant compound present in CKI, has also been shown previously to perturb a number of signaling pathways [16] and alter/arrest the cell cycle in a variety of cancer cells [17–21] and we have confirmed this observation (Fig. 4a) in Hep G2 cells. Our results permit direct comparison with CKI because our experiments have been done using equivalent concentrations of oxymatrine alone or in CKI. While oxymatrine has an effect on the cell cycle, it is not as effective at perturbing the cell cycle as is CKI. This indicates that oxymatrine must interact with other compounds in CKI to have a stronger effect on the cell cycle.

### Energy metabolism suppression by CKI

We have shown for the first time that CKI can inhibit energy metabolism as demonstrated by lower levels of NADH/NADPH and a higher [ADP]/[ATP] ratio. These results, combined with lower glucose utilisation and higher lactate levels indicate that this suppression was likely due to inhibition of the TCA cycle or oxidative phosphorylation. Previously, Gao et al. [7] have reported that CKI significantly increased the concentration of pyruvate in the medium and this observation in combination with our results supports a decrease in metabolic flux through the TCA cycle as the likely cause of the reported suppression of energy metabolism. Interestingly, oxymatrine on its own had the opposite effect on [ADP]/[ATP] ratio compared to CKI, indicating that it can enhance energy metabolism and increase the energy charge of the cell.

### DNA repair suppression by CKI

There is only one report in the literature of oxymatrine inducing DSBs [22] and no reports with respect to CKI. Our results show for the first time that not only does CKI induce DSBs, but that is also likely inhibits DNA repair by decreasing the expression of the Ku70/Ku80 complex required for NHEJ mediated DNA repair. It is worth noting however, that the reported effect of oxymatrine on DSBs [22] uses significantly higher (4–8 fold) concentrations of oxymatrine compared to our experiments. In our hands oxymatrine alone at 0.5 mg/ml showed no effect on DSBs as judged by the level of *γ*-H2AX after 24 or 48 h.

## Conclusions

**Fig. 5:**
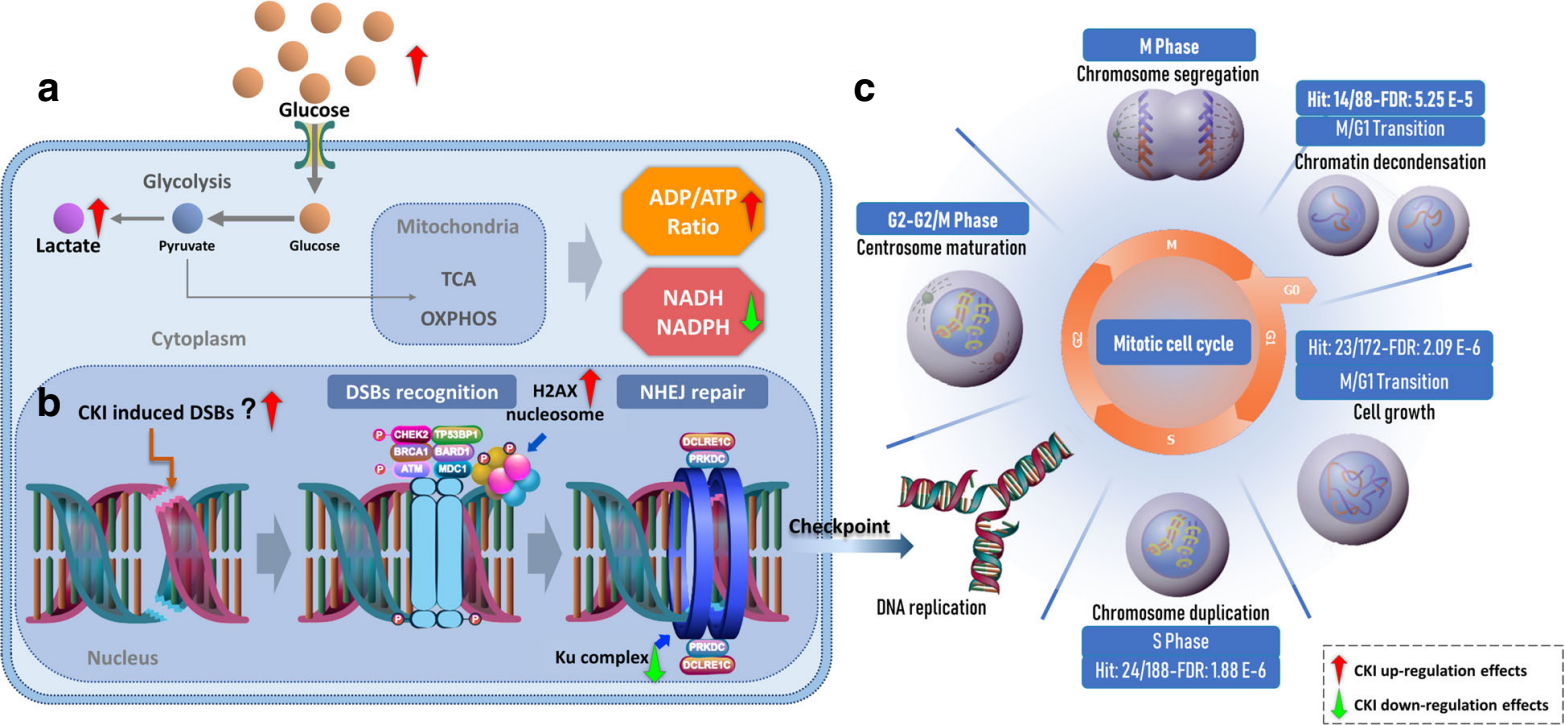
Integration of the three pathways altered by CKI. **a** General presentation of energy metabolism affected by CKI. Glucose utilisation is down-regulated by CKI. This is accompanied by increased lactate in the cytoplasm as CKI inhibits glucose metabolism downstream of glycolysis, leading to an increase in [ADP]/[ATP] and decrease in NADH/NADPH. **b** Effects on DNA repair in cancer cells by CKI. CKI may be able to directly induce DSBs, but may also indirectly induce DSBs by arresting checkpoint functions during the cell cycle. In addition, CKI may also inhibit NHEJ, the major repair mechanism for DSBs. **c** Reactome functional enrichment of cell cycle genes based on shared differentially expressed (DE) genes from previous studies. From M/G1 to S phase, the shared DE genes from both cell lines were significantly enriched. Most of these DE genes, were down-regulated

CKI causes suppression of energy metabolism and DNA repair along with altered cell cycle (summarized in Fig. 5). CKI has also previously been reported to induce apoptosis in cancer cells [8]. The overarching question is if CKI has independent effects on these three pathways or if the primary effect of CKI is through a single pathway that propagates effects to other, physiologically linked pathways. It may be that CKI suppresses energy metabolism thus disrupting downstream, energy hungry processes such as DNA replication and DNA repair. Alternatively, there could be independent effects on DNA repair leading to checkpoint induced cell cycle perturbation/arrest. Our results based on oxymatrine treatment of Hep G2 cells indicate that the cell-cycle is likely directly affected by oxymatrine and thus CKI. However oxymatrine alone had no effect on DNA repair and boosted, rather than reduced the energy charge of the cell. Taken together, these results support a model of many compounds/many targets [23] for the mode of action of CKI, where multiple compounds affect multiple targets and the synergistic, observed effect is significantly different to that seen with individual components.

## Additional file

Additional file 1 Contains supplementary figures as referred to in the main body of the paper. (PDF 721 kb)

## Abbreviations

CKI: Compound Kushen injection
CMM: Chinese meteria medica
DE: Differentially expressed
DSBs: Double strand breaks
Ku70/Ku80: The Ku heterodimer proteins
NHEJ: Non-homologous end joining
TCM: Traditional Chinese medicine
