# Supplementary material for: Cell cycle, energy metabolism and DNA repair pathways in cancer cells are suppressed by Compound Kushen Injection
Source: BMC Cancer. 2019 Jan 24;19:103. doi: 10.1186/s12885-018-5230-8 (PMC6345000; doi:10.1186/s12885-018-5230-8)
Supplement: Supplementary file 1 — Contains supplementary figures as referred to in the main body of the paper. (PDF 721 kb) [file 12885_2018_5230_MOESM1_ESM.pdf]

Additional File1:
Cell Cycle, Energy Metabolism and DNA Repair Pathways in Cancer Cells are Suppressed by Compound Kushen Injection

Jian Cui, Zhipeng Qu, Yuka Harata-Lee, Thazin Nwe Aung, Hanyuan Shen and David L Adelson

The University of Adelaide, School of Biological Sciences, Dept of Molecular and Biomedical Sciences

1 SUPPLEMENTARY DATA

1.1 Methods

XTT assay: The wells of 96-well tray were seeded with 4×10^3 cells per well for Hep G2 cells and 8×10^4 cells per well for MDA-MB-231 cells in 50 μL of medium and cultured overnight. On the following day, 50 μL of either medium, CKI or 5-FU were added to the cells. Viability of the cells was measured at 0, 24 and 48 hours after the treatment by adding XTT:PMS (50:1; Sigma-Aldrich). After 4-hour incubation at 37 °C optical density (OD) of each well was read at 490 nm. The background OD was also measured and the average was subtracted from the OD readings of appropriate wells.

1.2 Figures

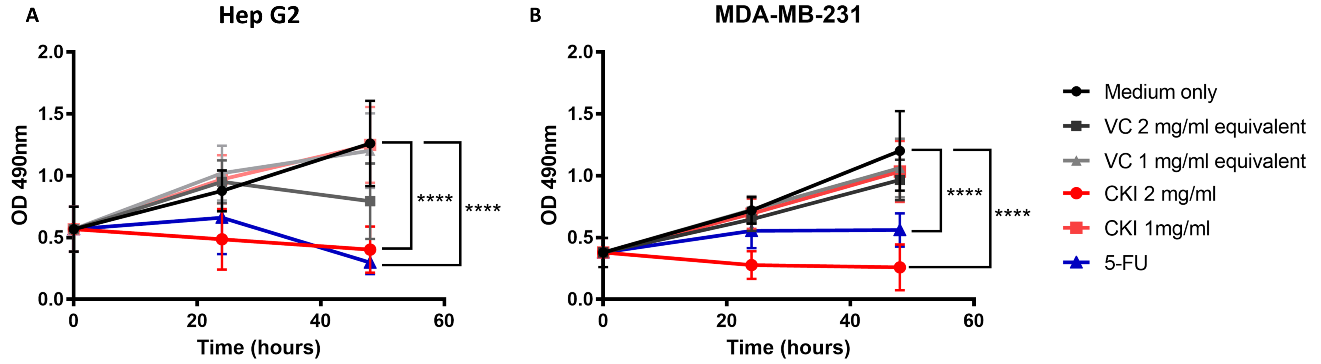

Figure S1. XTT assay result of Hep G2 and MDA-MB-231 cell lines. The XTT assay measures levels of NADH and NADPH by producing a formazan dye product that can be detected at 490nm. **A**. XTT assay result for Hep G2 cells. The assay was carried out at three time points: 0, 24, and 48 hours. 5 treatment groups were used and compared, 150 μg/ml 5-FU as a positive control for a cytotoxic agent, 1 mg/ml and 2 mg/ml CKI as well as the corresponding concentration of vehicle control (VC). CKI has a clear effect on the amount of formazan dye produced indicating a significant and marked suppression in the production of NADH and NADPH. **B**. XTT result of MDA-MB-231 cells. This test is with a low concentration of 5-FU (20 μg/ml). CKI has a clear and marked effect on the level of formazan dye produced indicating a significant and marked suppression in the production of NADH and NADPH. Statistical analyses were performed using two-way ANOVA comparing with untreated (****$p<0.0001$); bars show 1 standard deviation from the mean.

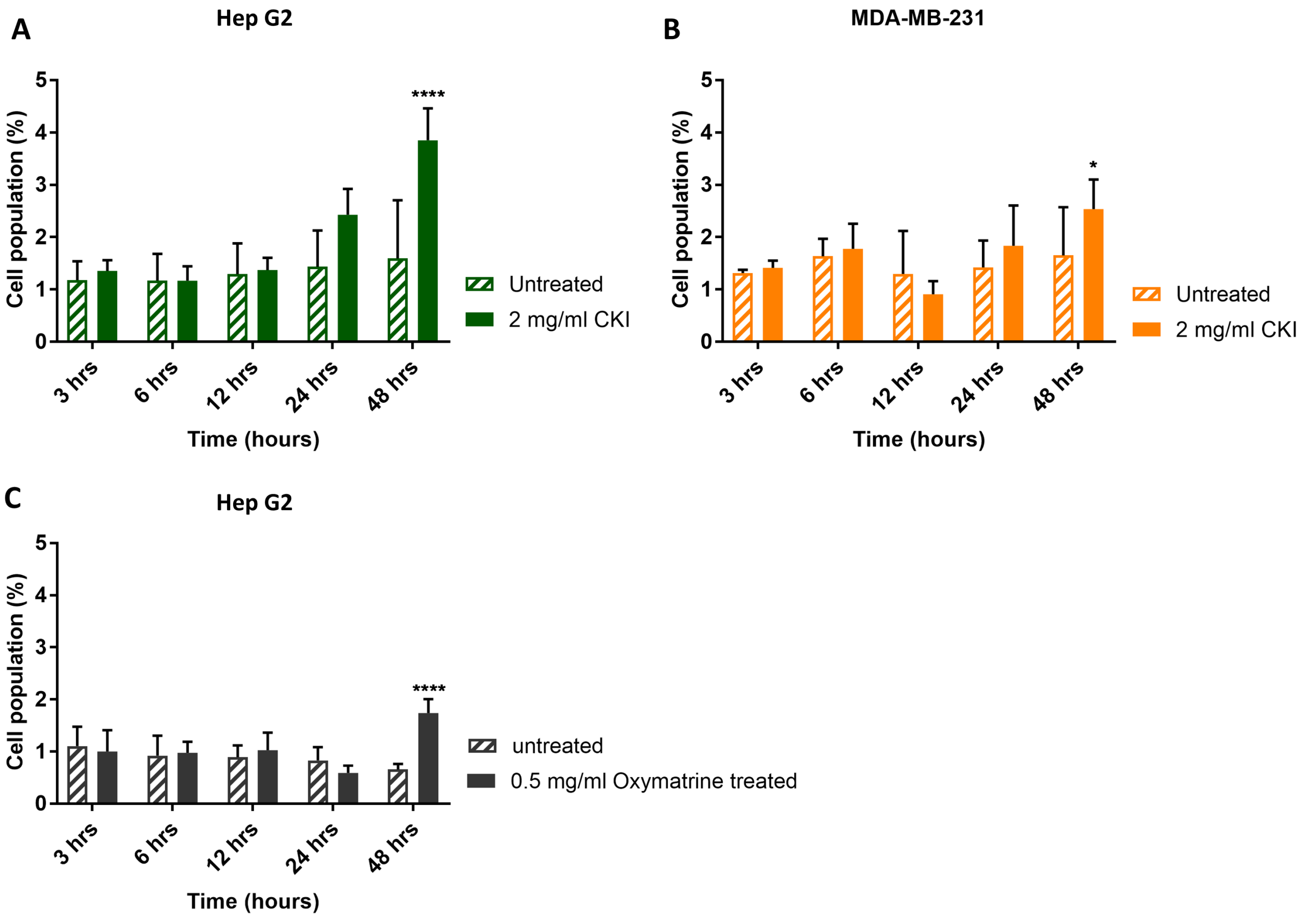

Figure S2. Cell apoptosis assay. A. Cell apoptosis in Hep G2 cells treated with CKI. The assay was carried out at 5 time points to detect apoptosis levels between untreated and 2 mg/ml CKI treated groups. From 3 to 12 hours, both groups maintained a baseline level of apoptosis. After 24 hour, apoptosis of CKI treated cells increased, with the difference attaining statistical significance at 48 hours. **B**. Cell apoptosis in MDA-MB-231 cells treated with CKI. From 3 to 24 hours, both groups show similar, if noisy results. By 48 hours apoptosis has increased and was statistically significantly different to the control. **C**. Cell apoptosis in Hep G2 cells treated with oxymatrine. We compare apoptosis levels between an untreated group and a group treated with 0.5 mg/ml oxymatrine. From 3 to 24 hours we observed a baseline level of apoptosis. By 48 hours apoptosis in the oxymatrine treated group is significantly greater than in the control group. Statistical analyses were performed using two-way ANOVA comparing with untreated (****$p<0.0001$); bars show 1 standard deviation from the mean.
